# Characterization of Cell Scaffolds by Atomic Force Microscopy

**DOI:** 10.3390/polym9080383

**Published:** 2017-08-21

**Authors:** Jagoba Iturri, José L. Toca-Herrera

**Affiliations:** Institute for Biophysics, Department of NanoBiotechnology, University of Natural Resources and Life Sciences, Muthgasse 11, 1190 Wien, Austria; jose.toca-herrera@boku.ac.at

**Keywords:** cell scaffolds, atomic force microscopy

## Abstract

This review reports on the use of the atomic force microscopy (AFM) in the investigation of cell scaffolds in recent years. It is shown how the technique is able to deliver information about the scaffold surface properties (e.g., topography), as well as about its mechanical behavior (Young’s modulus, viscosity, and adhesion). In addition, this short review also points out the utilization of the atomic force microscope technique beyond its usual employment in order to investigate another type of basic questions related to materials physics, chemistry, and biology. The final section discusses in detail the novel uses that those alternative measuring modes can bring to this field in the future.

## 1. Introduction

**Figure 1:**
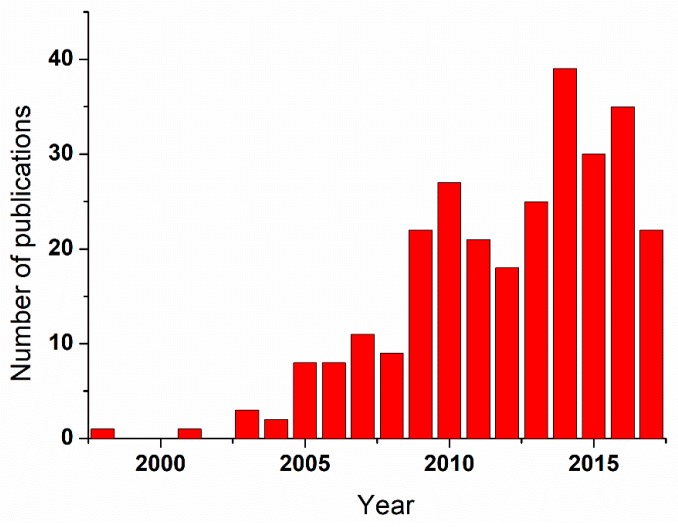
Time evolution of the number of published works within the field of cell scaffolds combined with Atomic Force Microscopy.

Although the first articles date from the mid-late 1990s and early 2000s [1,2,3], a quick search by Scopus database for the terms “Atomic Force Microscopy (AFM) + scaffolds + cells” brings, interestingly, only around 300 articles referring to them. From these results, it can be deduced that the field has still a long way to go. In the same line, the trend followed by the number of publications all along these years speaks about a timid periodical increase in 5 years periods that, even though it does not seem to definitely take off, stays as a very promising topic (see Figure 1).

The design and development of cell scaffolds for tissue engineering purposes has undeniably gained, on its own, more and more relevance in the last 30 years. The required biocompatibility of the materials employed for constructing the scaffolds, accompanied by their ease for being either degraded or metabolized, has become a challenge for researchers in both the biomedicine and materials science fields. Scaffolds have to provide an architecture on which seeded cells can organize and develop into the desired organ or tissue prior to implantation [4]. By means of this tissue engineering process the risks of immunological responses (rejections or viral infections) are certainly decreased and almost supressed. Among the requirements that scaffolds should fulfil for having an optimal function, they must pay attention to their topography and mechanical properties, factors which undoubtedly influence the way cells proliferate, organize, and give rise to a new tissue [5,6,7,8].

In this regard, atomic force microscopy is a well-suited and popular technique to analyze topographical features and mechanics at the nanoscale. Up to date AFM has been widely used, due to its unmatched resolution, in material science, physical chemistry, chemical physics, surface science, and almost all the fields related to the molecular scale [9,10,11]. However, over the last 20 years AFM has also become a standard tool in life sciences for studying biological phenomena, varying from surface topography [12,13,14], to structural studies [15], to the quantification of interactions between biomolecules [16,17]. Then, AFM results in a highly valuable complement to other characterization techniques currently employed by the cell scaffolds community.

In this document, we perform a brief overview of the different published results that have covered so far this exciting topic of AFM together with scaffolds of a different nature, and of the way these were experimentally obtained. Special attention is paid to the material/system forming the matrix of the scaffold, as well as to the AFM measuring mode employed. For the reader’s guidance, the covered scaffold examples have been divided, merely attending to the authors’ personal criteria, into five major topics. The first four represent very general systems, where the usage of quite standard polymers as building blocks (i.e., polyurethane or polylactide) is in a majority. The fifth group, in turn, presents the use of diverse biopolymers and biomolecules, either with the Arg-Gly-Asp tripeptide (commonly known as RGD) moieties or without them. The decision to make a group apart with them derives from their capability, in some cases, to undergo self-assembly, together with their unique conditions for specific cell recognition. By the mentioned spontaneous arrangement they are capable of forming diverse structures that would hardly fit into one of the other categories exclusively. In turn, the focus of AFM measuring modes has principally been set on the different mechanical studies covered by the literature rather than on topography imaging, since the former are less extended and well-known. These mechanical analyses of cell scaffolds include elastic moduli, adhesive properties, and more sophisticated analyses like single chain stretching and/or the so-called single-cell probe force spectroscopy, where a living cell acts as a measuring probe, allowing quantitative characterization of what the cell-to-scaffold affinity can be. Bibliographic references regarding all these systems and measuring methodologies will be covered in this report. Furthermore, for the sake of a better understanding of these concepts, we will very briefly introduce the atomic force microscope as both an imaging and a mechanical machine, and discuss different methods that might be future interest for the community.

## 2. Atomic Force Microscopy, a Versatile Tool

Since its appearance in the mid-80s, in consequence of the first probe microscopes developed [18], atomic force microscopy has become a very popular and useful tool to characterize matter at both the nano- and micro-scale levels. Through the years, together with its continuous evolution and technical implementation, AFM has shown high versatility and an almost on demand adaptive capability. The systems covered in this 30-year range, i.e., from Deoxyribonucleic acid (DNA) strands up to eukaryotic cells, traversing many soft/hard materials, have contributed to its extended reputation and its presence in many (nano) biotechnology laboratories. Neverthless, its potential use is still sometimes underestimated and certainly limited to only a few of its operating features. We then consider it crucially important to emphasize the role that this technique could play, in co-operation with other complementary techniques, in a field of growing interest like that of cell scaffolds.

### 2.1. AFM as an Imaging Machine

**Figure 2:**
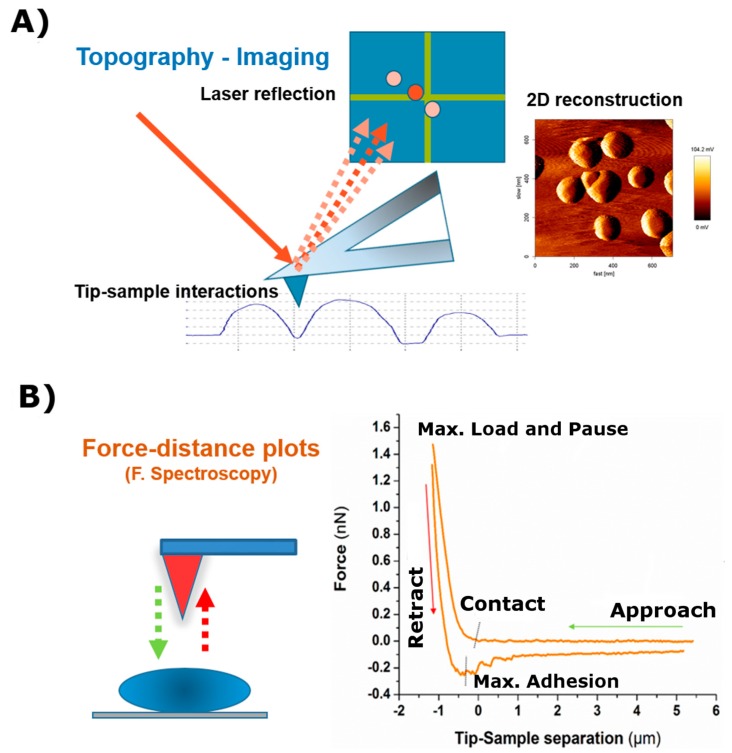
Scheme showing the main measuring modes in Atomic Force Microscopy. (**A**) Imaging mode and (**B**) Force Spectroscopy mode.

Atomic Force Microscopy has proven to be a solid alternative to other microscopy techniques (i.e., transmission and scanning electron microscopes) when investigating nano-scale structural features of (bio)materials, under an aqueous environment and at different temperatures [19,20]. In the current case, the sensing element is a flexible cantilever with a sharp tip at the end, as shown schematically in Figure 2A. The deflection of this cantilever is brought by tip-sample interaction and yields a continuous shift in the reflected laser beam, as measured with a position detector (photodiode). In addition, a piezo scanner moves the cantilever along the three dimensions. The topography of the sample is thus obtained from translation of the tip/sample interaction-derived voltage variation, and the final image is delivered by computer processing. 

The most popular ways of obtaining topography imaging are contact and tapping modes. In contact mode, the value of the repulsive force between tip and sample is kept fixed during the scanning of the sample. In tapping mode, in turn, the cantilever oscillates at its resonant frequency (or close to) and when approaching the sample, the tip comes into intermittent contact with the surface. This induces a reduction in the amplitude of the oscillations resulting from the tip-sample interactions. The amplitude is then used as a feedback signal for topographic imaging. In this way, by recording the difference between the phase of the (set) drive signal and the phase of the cantilever response, a “phase” image is obtained. For instance, this type of imaging has been used to deliver information about the viscoelastic and adhesive properties of the sample [21,22] or, more recently, to resolve 3D structures formed by electrolyte solutions near a solid surface [23]. The tapping mode, in comparison with the contact mode, presents the advantage of reducing friction forces when scanning (soft) samples. In any case, the scanning force should be always kept as low as possible to avoid sample damaging, unless required, which implies the use of cantilevers of low (<0.1 N/m) spring constant values. Furthermore, the current commercial AFM design and the available thermally-controlled fluid cells allow measuring at 37 °C in different buffer solutions, in order to mimic body conditions and provide a stable system for biological samples.

### 2.2. AFM as a Mechanical Machine

Imaging is not the only feature from atomic force microscopes, despite its extended use. AFM devices can also be used as a “mechanical” machine allowing the investigation of adhesion and surface forces [24,25], polymer elasticity [26,27], ligand-receptor forces [28,29], or cell mechanics [30,31,32,33]. In these experiments, an AFM-tip or a colloidal probe [34] is extended and retracted towards/from the sample of interest by following exclusively the Z-axis. Such motion takes place under controlled displacement speeds, as governed by the movement of the piezo. During this process, the deflection of the cantilever is determined as a function of the displacement of the piezo-scanner [20], and the force sensed by the cantilever is calculated using Hooke’s law (which is equal to the cantilever deflection times of its spring constant). Therefore, the spring constant of the cantilever should be calculated in every experiment. A review of the different experimental calibrating methods can be found in [35].

The force-distance curves recorded in this way (Figure 2B) can be divided into three clearly distinguishable segments (approach, contact with the sample, and retraction). They can be described as follows:

- The approach curve delivers information about the existing repulsive or attractive forces between the tip/colloidal probe and the sample (e.g., electrostatic, van der Waals, hydration, or entropic forces). These type of measurements have been crucial for the understanding of molecular and colloidal interactions [11,36,37,38].
- The second part of the curve, during contact between the cantilever and the sample, provides information about rheology-related properties (e.g., Young´s Modulus, stiffness, relaxation time, and viscosity). The estimation of both the sample stiffness and elastic modulus (E) has been described in [39,40]. In this regard, the Hertz model—in which the contact between two linear, elastic spheres is described—is one of the most commonly used models to calculate the Young’s modulus from an AFM force-distance curve [41]. However, this model presents some limitations, mostly related to the omission of the adhesive forces, which limits its applicability on sticky materials. Alternatively, the Derjaguin-Mfiller-Toporov (DMT) and Johnson-Kendall-Roberts (JKR) theories were developed to overcome such limitations [42]. Also, the employment of indenter geometries different than spheres has given rise to additional adjustments, as with the Sneddon model, which is applied for conical shapes [43] and the derivative equations developed for quadratic pyramids or flat indenters [44,45,46]. In addition, more information can be obtained by keeping close contact between the tip and the material for a certain observation time (*t*_observation_), which is normally denoted as the Dwell time. Depending on the measurement performed, either the Z position of the head (Relaxation) or the load applied (Creep Compliance) are fixed during the contact. This induces the material to undergo structural rearrangement in response to the load-induced deformation which, by extension, allows the obtaining of the compressive moduli and viscosities of the material tested [47].
- Finally, the segment depicting the retraction motion relates to adhesive forces, the existence of tethers, and possible molecular unfolding events. The maximum adhesion (*F*_adh_) parameter, or pull-off force, is indicative of the stickiness of the sample. It is brought by the minimum of the peak in the retraction segment. Additional pulling shows the recovery path followed until achievement of the non-contact state. This tip-sample retraction can take place either via tether formation, in the shape of uniform rupture events distanced by plateaus of zero force variation [48], or when capturing individual molecules/chains, by means of saw-like adhesion peaks to be fitted by a worm-like chain (WLC) model [49,50].

It is worth mentioning that any interaction force depends on the distance between the interacting bodies. The same applies for the estimation of the indentation caused by external pressure. Therefore, an optimal detection of the contact point between tip and sample favors quantification of the type of interaction or physical quantity considered. In order to solve such an issue, it is worth highlighting the work of Benitez et al. with R programming language [51], in which an algorithm covering the statistical analysis of the slope changes of the curve is presented.

## 3. AFM and Cell Scaffolds: Literature Review

Based on the aforementioned measuring methods, some authors in the field of cell scaffolds have already explored the usefulness of Atomic Force Microscopy for testing different featuring aspects of their engineered materials. A broad majority of these studies obtained an advantage of the topography imaging aspects of the technique. In them, the reported research showed the analysis of a large range of materials such as natural and synthetic polymers [52,53,54], collagen [55], silk fibroin [56,57,58], cellulose [59], peptides [60,61], graphene and carbon nanotubes (CNT) [62,63], particles [64,65], or patterned structures [66,67]. This list, kept short for compact design reasons, could include more field-related articles. However, the ones here cited can undoubtedly be considered an optimal representation of each of the systems shown. A more detailed description of the cases commented has been included as a Supporting Material (Table S1).

In the following paragraphs, the description will be focused on those research works offering a more alternative use of the AFM technique, in which characterization is tackled from the point of view of mechanical properties. As already explained in the Introduction section, the studies under consideration have been divided into five main sections, attending to the matrix forming the scaffold, and are presented as follows:

### 3.1. Fibres

The group of fibre-like cell scaffolds is the one that presents, by far, the largest number of contributions. In the recent years, through the development of new materials and the re-definition of some existing techniques, i.e., electrospinning, researchers have found an easy way of fabricating nanometric fibres of diverse composition and multiple potential applications. In this process, AFM has become very useful in order to perform different mechanical characterization tests, ranging from classical nanoindentation up to the trapping and stretching of individual fibres, which leads to discrimination between segments of the force-distance plot required for the respective analysis.

Thus, Dunne et al. (2014) performed indentation studies (approach segment) to determine the apparent elastic modulus of dielectrophoresis-aligned nanofibrous silk fibroin-chitosan scaffolds under alternating current frequencies and the presence of ions [68]. The same protocol was subsequently followed to assess the apparent elastic modulus of human umbilical vein endothelial cells (HUVECs) seeded onto these type of scaffolds. Horimizu et al. (2013) went for thick periosteal sheets with enhanced cell layering [69]. Nano-indentation by AFM allowed distinguishing between the central and peripheral regions by means of the peak stiffness values of cells. In a similar manner, Liu et al. (2014) compared extra- to intra-fibre-mineralized collagen scaffolds. They showed how these two systems exhibit a different nanostructure. Furthermore, the intra-fibrillar-mineralized scaffold, presenting a bone-like hierarchy, was featured by a significantly increased Young's modulus in both dry and wet conditions if compared to the other one [70].

The retract segment was also used by Guarino et al. (2016) to characterize the influence of hydroxyapatite crystals on PCL composite scaffold properties with relevant effects on their biological response [71]. Hence, AFM yielded a significant increase of the pull-off (adhesion) forces from 33.7% to 48.7%, and almost a two-fold increase in roughness, as determined by imaging. These results could be also compared to complementary differential scanning calorimetry (DSC) analyses indicating a reduction of the crystallization heat, from 66.75 to 43.05 J·g^−1^. An analysis of the adhesive properties was also used by Firkowska et al. in other alternative, fibre-like scaffolds like that of the multi-walled carbon nanotube (CNT)-based scaffolds [72]. This system represents a novel type of matrix composed of layer-by-layer assembled polyelectrolytes together with crosslinked carbon nanotubes with regular nano-topography. AFM investigation of the adhesion behaviour of the attached cells on the films assessed their cytocompatibility.

**Figure 3:**
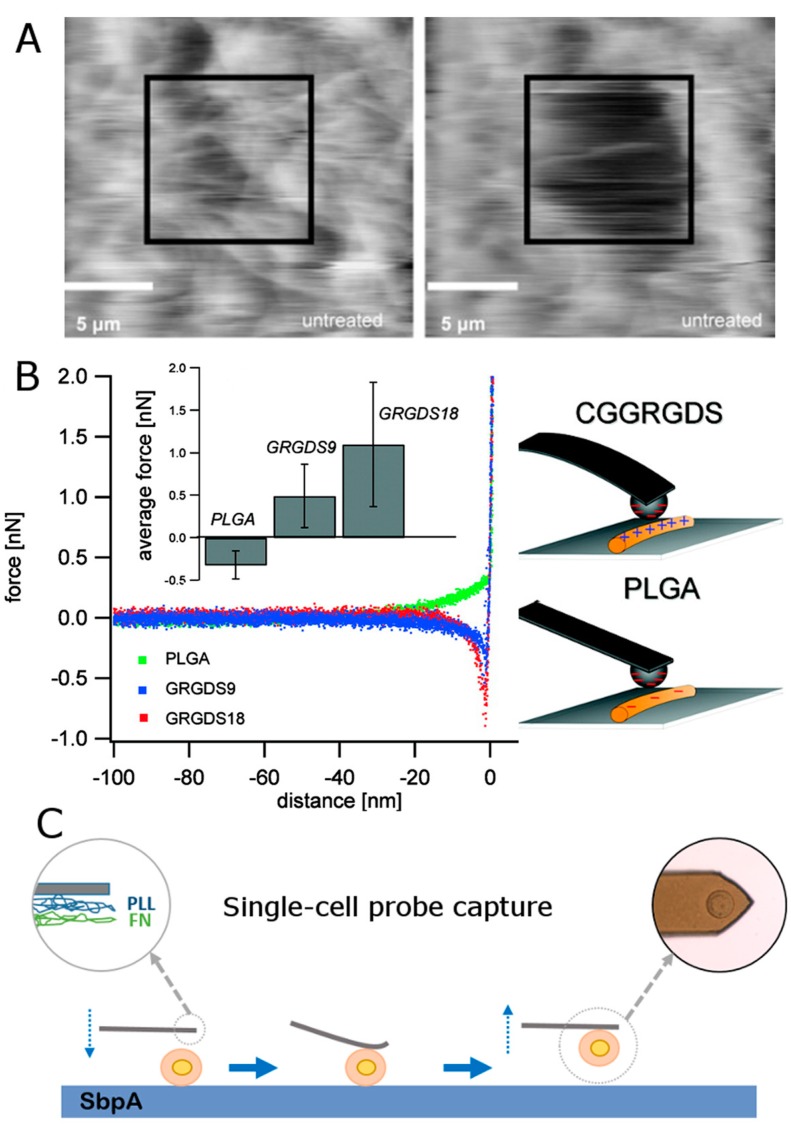
(**A**) Topographic scanning of collagen fibrils before (left) and after (right) scanning a 10 μm × 10 μm area at 5 nN. Scale bar represents 5 µm. Black boxes indicate the region where the increased normal force was applied. Imaging was performed under phosphate buffer at 1 nN loading force (Adapted reprint with permission from [75]. Copyright (2011) American Chemical Society). (**B**) Force−displacement curves resulting from colloidal interaction probing with a silica bead on (Gly-Arg-Gly-Asp-Ser) (GRGDS)-functional fiber compared to poly(lactic-*co*-glycolic acid) (PLGA) fiber, as schematically shown on the right sketch. The inlet shows adhesion force values as a function of PLLA-*b*-CGGRGDS content (0, 9, and 18 wt %) (Adapted reprint from [92], Copyright (2011), with permission from Elsevier). (**C**) The cell attachment process performed on an S-layer film. The fibronectin-functionalized tipless cantilever approaches towards the substrate and keeps contact for 10 min. Afterwards, the cantilever retracts slowly from the surface with the selected cell attached to it (an optical photograph shows the cell attached to the cantilever) (Reprinted from [93] with permission from the authors).

In a higher level of refinement and complexity, the experiments done by Baker et al. included a combined atomic force microscope (AFM)/optical microscope setup to study the mechanical properties of individual electrospun fibres. These were made of either poly-ε-caprolactone (PCL) (440–1040 nm of diameter) [73] or fibrinogen (diameter = 30–200 nm) [74]. The AFM was used to stretch individual fibres and to monitor factors such as elasticity and extension capability under both dry and wet conditions which, as the authors claim, might provide enough mechanical data to guide the construction of scaffolds and other biomedical devices based on these components. Such fine control over the tip location was also employed by Spurlin et al. (2009) for absolutely different purposes [75]. In their case, the AFM tip was applied over a defined region of enzyme tissue transglutaminase 2 (TG2)-treated fibrils in order to perform loading and shearing force studies. Results indicated a 3-fold higher resistance of treated vs. untreated fibrils, which apparently alters the contractile and proliferative response of the material. The perturbation produced can be seen in Figure 3A.

### 3.2. Patterned Structures

The last publication mentioned above, where the AFM tip is locally utilized to produce structural changes through shear forces under high loads, opens the path to introduce the use of this technique into another group of systems, that of patterned surfaces. Hence, by means of controlling both the force applied and the exact location of the interacting tip, researchers have already envisaged the use of force microscopy to induce the formation of well-defined patterns.

One could, for instance, employ the tip as a lithographic crayon. This was presented by Podestà et al. (2005) in order to design chemical and morphological micro- and nanoscale modification of poly(2-hydroxyethyl methacrylate) (PHEMA) hydrogels produced by conventional radical polymerization [76]. In their work, the lateral motion of the tip, in combination with a high load, is applied to create different topographies by scratching the underlying polymer surface both in the presence and the absence of carbon nano-particles. Another approach was that applied by D’Acunto et al. (2007) to create patterned polymer films with tailored length scales [77]. Thus, the morphology of poly(ε-caprolactone) diol (PCL) and poly(ethylene terephthalate) (PET) is changed in the shape of ordered ripple structures by following a novel methodology, just by a single AFM scan and for relatively low applied loads. As explained by the authors, such ripple structures can be modulated and modified by changing the applied load, scanning velocity, and angle. In this way, it is possible to obtain sinusoidal structures with suitable amplitude, periodicity, and orientation. Additionally, the method is claimed to be used for the nano-patterning of large areas making it of high usefulness in many applications.

Of course, besides the more specific uses as those listed above, other researches utilize AFM in a more standard mechanical way by performing nanoindentation on the sample. This is the case of the work of McCracken al. (2016) who determined the mechanics of different PHEMA-based 3D hydrogel cellular microcultures, under different curable monomer compositions, and which are prepared via direct ink writing [78]. Their observations revealed that depending on the polymer to monomer ratios (*M*_r_), the prepared inks yielded mesh gels of a different opening due to a lower degree of entanglement which, by extension, could impact the mechanical performance of the films. In turn, Park et al. (2015) analysed the mechanical responses of cancer cells on top of the nano-scaffolds employed to control their adhesion size [79]. On the one hand, they found that prostate cancer cells showed a linearly decreasing proliferation rate and mechanical stiffness as the size of the imprinted nanoislands decreased. This mechanical signature was exacerbated for less metastatic prostate cancer cells. On the other hand, breast cancer cells showed no dependence of mechanical responses on the geometric properties of the nano-scaffolds, despite the acute inhibition of adhesion and the abrupt mechanical changes.

### 3.3. Particles

Among the materials employed for cell scaffold fabrication, (nano) particles are not as extended as other building blocks but still have found their own space. It is pretty common to find them as part of composites, as shown by Yang et al. (2016) for the nanofibers of silk fibroin (SF) with up to a 40 wt % of uniformly dispersed hydroxyapatite particles (HAp) in their composition [80]. The dependency of the mechanical moduli on the content of the HAp nanoparticles was analyzed at the AFM using a three-point bending method by means of a tipless cantilever. The SF/HAp composite fibers at varying mixing ratios were electrospun over micrometer scale channels, and so the suspended SF/HAp nanofibers could be gently pressed at a controlled speed to record the corresponding forces and deflections at each location. Based on the force and deflection data, authors calculated the bending modulus of each of the SF/HAp composite nanofibers. From their results, single nanofibers became stiffer with a higher content of the HAp nanoparticles up to 20 wt % of Hap, while the further addition of the HAp nanoparticles reduced the mechanical strengths.

But particles can also be employed as an individual entity to build a scaffold. This is the case of the work published by Hong et al. (2010) [81], where mono-dispersed bioactive glass nanospheres (200–350 nm in diameter) were shown to induce rapid deposition of an apatite layer in simulated body fluid. This speaks about their excellent bio-mineralization capability. Then, authors used AFM to investigate the effect of such bioactive glass on the biomechanical properties of various mammalian cells, such as bone marrow stem cells and/or bovine aortic endothelial cells seeded on top, with diverse results depending on the cell line.

### 3.4. Hydrogels

In a similar level to fibrous scaffolds, hydrogels represent a largely exploited system for the design of cell scaffolds. These soft polymeric networks, of either natural or synthetic origin, feature by having a huge water content which turns them into a perfect model for biological tissue mimicking [82]. However, those factors contributing to their good properties as supporting scaffolds are not beneficial for obtaining an optimal high-resolution characterization of their structure by AFM. For instance, sample softness and high stickiness represent an extreme limitation when performing topographical imaging. It is then when the Force Spectroscopy mode becomes of primary importance, so the mechanical behaviour of the scaffold can be determined and then combined with other visualization techniques.

Abuelfilat et al. (2015) employed Scanning Electron Microscopy (SEM)-based X-ray ultramicroscopy (XuM) to visualise, reconstruct and analyse 3D porous structures of hydroxypropyl cellulose methacrylate (HPC-MA) hydrogels [83]. Then, by incorporating AFM measurements, the elastic modulus of the hydrogel was determined, and mechanical modelling of individual pores and the bulk scaffold also proved to be feasible. In similar terms, Credi et al. (2014) measured mechanical properties of divinyl sulfone (DVS)-crosslinked hyaluronic acid hydrogels [84]. By varying both DVS content and curing time, the control over the crosslinking process can be achieved in order to prepare biocompatible hydrogels with mechanical properties closely approximating those of the extracellular matrix (ECM) of natural stem cells niches.

One of the rare cases in which AFM could also deliver images of the surfaces topography was presented by Ohya et al. (2005) for Poly(*N*-isopropylacrylamide) (PNIPAM)-grafted gelatin hydrogel surfaces, a temperature-induced scaffold at a physiological temperature [85]. In this study, the authors aimed at determining the effect of the graft architecture of thermoresponsive PNIPAM-gelatin on the surface topography and elastic modulus of the hydrogels. Also, mechanical properties were determined by AFM.

### 3.5. Peptides and RGD Sequence

Last but not least, the fifth group in which scaffolds have been divided is devoted to peptides and assemblies containing RGD derivatives. As already mentioned above, the particularity of these biopolymers turns them into an own entity, not really matching into any of the already listed ones but, at the same time, being part of all of them. The best example of this observation is that of self-assembling peptides, which have been reported to form either well-defined fibrous structures [61,86] or more hydrogel-like films [87,88]. An additional interesting feature of peptides, and more specifically those containing RGD-moieties, is the capability of inducing specific recognition of cells based on the selective capture of integrins forming part of their Extra-Cellular Matrix (ECM) [89]. This factor has promoted the development of a large number of research studies in which the binding properties of either fibronectin, fibrinogen, or alternative synthetic peptide chains with the RGD sequence in their structure have been exploited.

In many cases, such peptides are easily combined with another matrix, like polymers, in order to activate the cell capture of systems that otherwise would remain almost inert [90]. Sabater i Serra et al. (2016), for instance, assembled fibronectin (FN) on top of 2D Poly(ethyl acrylate) (PEA) films [91]. These could be tuned by varying amounts of a crosslinker (1–10% of ethylene glycol dimethacrylate). Even though PEA has a good ability to organize FN, under an increasing crosslinker density the segmental mobility was shown to be significantly reduced. In turn, Gentsch et al. (2011) presented a single step procedure to fabricate bio-functional fibres composed of the polymer- (Cys-Gly-Gly-Arg-Gly-Asp-Ser) peptide conjugate (poly(lactic acid)-block-CGGRGDS) combined with poly(lactic-*co*-glycolic acid) (PLGA) [92]. Authors then could test the surface accessibility of the peptide on the nanoscale by AFM measurements probing the electrostatic interaction between CGGRGDS surface functionalities and a colloidal silica probe via atomic force microscopy, and the corresponding comparison with pure PLGA fibres. A short scheme of the experiment is shown in Figure 3B. The repulsive forces of pure PLGA contrast with the adhesion measured for CGGRGDS-containing samples (9 and 18 wt %).

Regarding the use of integrin for specific recognition purposes, a very different approach can also be attempted: an integrin coating would activate AFM cantilevers (either with a tip or tip-less) so, under optimal and controlled contact maintenance, they can be used to bind and test living cells. Thus, this living cell would act now as a measuring probe. This new cell-cantilever joint system can be applied to measuring interactive forces with a surface/scaffold of choice, in a process known as Single-Cell Probe Force Spectroscopy (SCFPS). Of course, the success of the process also depends on the employment of a substrate of known low affinity towards cell binding, so cells stay as round and intact as possible before being captured. Such a type of experiment was presented by Moreno-Cencerrado et al (2016) [93]. They could design a new setup consisting of two different half-surfaces coated either with a recrystallized SbpA bacterial cell surface layer proteins (S-layers) or integrin binding Fibronectin, on which Michigan Cancer Foundation (MCF)-7 breast cancer cells were incubated (see Figure 3C). As shown in their work, quantitative results about SbpA-cell and Fibronectin-cell adhesion forces as a function of the contact time could be described by this technique. At the same line, Taubenberger et al. (2014) reported about a full bunch of alternative components and factors that might be considered when designing SCFPS experiments [94].

## 4. Conclusions and Outlook

The analysis of cell scaffolds by atomic force microscopy has gradually gained more and more relevance over the last years. The presence of an AFM at nearly all academic and research institutions has contributed to it favorably. However, it has to be said that the potential applications of AFM are still waiting to be used in research topics such as those discussed in this short review. As the reader can see in the previous sections, AFM has had a minor role so far, acting as a complement to other measuring techniques. Perhaps, a slight redefinition and description of its real potential uses may trigger its use in future studies.

For instance, most of the literature found merely regards topographical imaging of diverse systems. It is true that such a type of topographical characterization of scaffolds becomes important when studying samples in which a defined pattern or a crystal-like structure on the sub-micron scale is involved, but its interest drops significantly in the other cases. Indeed, scanning electron microscopy (SEM) could cover this part of the characterization of the sample of interest with similar results. One idea is to extend the classical concept of imaging (height, error deflection, and phase) by combining the raster scanning with force-distance curves. This is possible because the AFM functioning relies on the tip-sample interacting forces and, therefore, a topography image is already a result of those forces. Then, by considering this overall force-dependence concept, one can obtain and develop new imaging modes which depend on diverse but concrete interactions. Examples of it are Chemical AFM and the Fluorescence sensing approaches, in which the tip is employed for obtaining another type of material-derived information (e.g., surface chemistry, electrical and hydrophobic properties, etc.).

**Figure 4:**
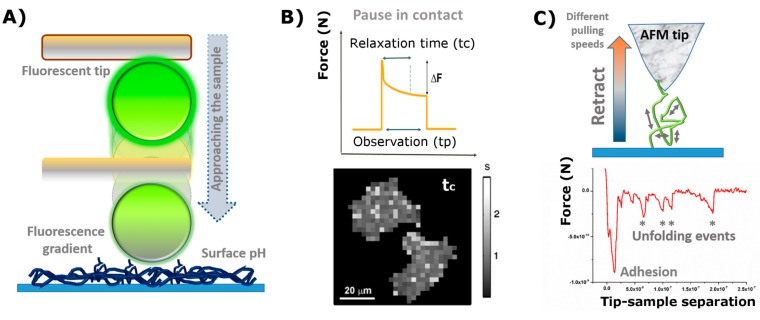
Potential uses of atomic force microscopy (AFM) according to the focus on each of the individual three main segments of a force-distance curve (note that time is an implicit variable in a force-distance curve and therefore can be also represented as a force-time curve). (**A**) Scheme of Fluorescence-based surface pH sensor mode, in which a stained colloidal probe is approached to the surface of interest. At sufficiently close distances the change in the pH induces quenching of the fluorescence emission (changes in surface pH as a function of time can be detected in “pause in contact mode”). (**B**) An example of Stress Relaxation imaging mode obtained on MCF-7 cells from mapping their relaxation time (*t*_c_) values, in seconds, according to the coloured scale on the right (*adapted reprint from* [97] *with permission of the authors*). The plot on top represents the usual shape obtained for a stress relaxation experiment, keeping the contact in constant height, where *∆F* denotes the force decay and *t*_p_ and *t*_c_ refer to the observation and relaxation times, respectively. (**C**) Scheme of a single chain pulling experiment. The pulling rate (speed) can be adapted to demand. Below, a representative retraction plot showing the most relevant unfolding events measured and the adhesion force peak.

At the so-called chemical force microscopy, the chemical modification of the tip (which can have different geometries), or even of the whole cantilever body if employing tip-less sensors, allows detecting specific and/or non-specific interactions when the tip is close enough to the sample (note that the forces involved are of the molecular or colloidal range). This is also denoted as molecular recognition [95,96]. If such chemical force microscopy is usually thought of for specific interactions, an alternative methodology that also explores the chemical environment of the sample of interest would consist of the use of fluorescent tips (or colloidal probes). The idea behind this would be to utilize a probe which is sensitive to pH changes. Hence, knowing the variation that changes in the pH induces in the emission the intensity of the fluorophore, as determined by a master curve, and the fluorescent tip could act as accurate pH sensor of the substrate. A schematic sketch is shown in Figure 4A. In this way it would be possible to have a full chemical map of the sample, attending to its surface pH, which in turn relates to its electrical properties (i.e., electrostatic surface potential).

However, besides the applications mentioned above, future predictions about the employment of AFM on cell scaffolds mostly stay on the side of the Force Spectroscopy measuring mode, either as a “single” or as a more “global” analysis. While the former does not deliver an image, the latter still allows the obtaining of a full mapping over the area of interest. In any of the cases, all the parts the force-distance plot is composed of (Figure 2B) should be taken into consideration for an optimal characterization. The usual nanoindentation experiments for determining the elastic modulus of the sample are sometimes not sufficient to complete the mechanical description of the sample. As explained in a previous section, extraction of the instantaneous Young’s modulus only corresponds to a partial analysis of the force-distance curve. The omission of the “pause in contact” and retraction directly discards a full bunch of parameters that could become a perfect complement for completing the characterization.

- The first part of the plot (approach motion) can be used for determining the presence of repulsive/attractive forces between the tip and the simple (electrostatic, steric, and/or entropic, etc.). Examples of it were already reported in the literature, among others, by Borkovek [24], and Melzak [98]. A similar process is also described by Gentsch et al. in reference [92], and depicted in Figure 3B.
- Attending to the contact segment of the plot (or pause in contact) or studying the deformation of the scaffold under constant force or stress relaxation experiments can describe the rheology of the material for sufficiently large observation (Dwell) times. This was successfully applied to characterize the response of breast carcinoma MCF-7 cells seeded on borosilicate glass substrates [97]. In their work, the authors also presented a new type of AFM imaging called Stress Relaxation imaging, based on the local relaxation times measured after subdividing the cell into multiple domains (mapping in Figure 4B).
- The retraction motion (third segment) yields, in turn, very useful information about adhesive forces and the work required to recover the non-contact state. A good example of it would be the aforementioned “single” force microscopy mode for the stretching of individual coiled chains adhered to the tip (as in the case of proteins) [99,100,101]. Hence, quantification of the occurring intermediate rupture events, as the cantilever moves perpendicularly away from the surface, might deliver a precise fingerprint of the forces which govern the internal arrangement of the coils (Figure 4C).

Latest developments have also considered the use of dynamic AFM methods (based on the tapping mode) to promote the nanoscale characterization (topographical and mechanical properties) of the cell scaffolds to another level. For instance, dynamic multi-frequency AFM methods (multi-harmonic, Bimodal) and Peak Force Tapping (PFT) are pushing the AFM field to a faster, quantitative, nanomechanical characterization of complex biological systems in a higher resolution [102,103].

These methods described so far imply the discriminate characterization of either the scaffold or the cells seeded on top. Nevertheless, it might also be very interesting to directly measure the interaction between the cells and the scaffold itself (which gives an idea about the compatibility cell-scaffold). This possibility is brought by the so-called Single-Cell Probe Force Spectroscopy (SCPFS). As previously described, it combines measurements in the Force Spectroscopy mode with chemical modification of the cantilever in order to capture living cells so they can be used as a measuring probe. SCPFS provides, as the main advantage, a sensitive approach to characterize not only the interaction of a whole cell with the underlying substrate but also that of single molecules, attending to host-guest interactions [88]. The versatility of this technique leans on the number of cell lines to be employed, either prokaryotic [61,90] or eukaryotic [84,89], and the infinite possibilities for scaffold design. A glimpse over the number of works published shows the increasing interest of the community into this methodology and, therefore, a promising future can be envisaged.

Almost at the end of this review, we could ask why AFM is not extensively used when investigating cell scaffolds. Or, what are the challenges that prevent AFM from becoming more popular in characterizing cell scaffolds? The reading of the manuscripts commented on and included throughout this review already reveal some hints to address such issue. Most of the researchers investigating cell scaffolds are not true AFM specialists. Our experience shows that the mastering of AFM requires at least: (i) very good experimental and technical skills; and (ii) a correct interpretation of the AFM results. This point is not trivial since it requires image processing to complicate calculations based on the physical models. Therefore, non-specialised AFM laboratories producing cell scaffolds might take a pragmatic approach by focusing mostly on the application and the technological effects of the scaffold (e.g., the influence on the cell fate). Our opinion is that in the years to come, a refinement in scaffold bioengineering will lead to a better understanding of the scaffold/cell interface and its corresponding biotechnological implications. Thus, a complete physico-chemical characterization either of the scaffold or the total system would be crucial, and AFM may be the right experimental tool to achieve that goal.

## Supplementary Materials

The following are available online at www.mdpi.com/2073-4360/9/8/383/s1, Table S1: Literature compilation regarding the exclusive use of Atomic Force Microscopy for topography imaging purposes.
