# Supplementary material for: Characterization of Cell Scaffolds by Atomic Force Microscopy
Source: Polymers (Basel). 2017 Aug 21;9(8):383. doi: 10.3390/polym9080383 (PMC6418519; doi:10.3390/polym9080383)
Supplement: Supplementary file 1 [file polymers-09-00383-s001.pdf]

Short Review – Supporting Information

Characterization of Cell Scaffolds by Atomic Force Microscopy.

Jagoba Iturri [1,*] and José L. Toca-Herrera [1]

[1] Institute for Biophysics, Dept. of NanoBiotechnology, University of Natural Resources and Life Sciences, Muthgasse 11, 1190 Wien, Austria; jagoba.iturri@boku.ac.at; jose.toca-herrera@boku.ac.at

* Correspondence: jagoba.iturri@boku.ac.at; Tel.: +43-1-47654-80339

Table S1. Literature compilation regarding the exclusive use of Atomic Force Microscopy for topography imaging purposes. The abbreviations of the experimental techniques correspond to AFM: Atomic force microscopy; CA: Contact angle; FM: Fluorescence microscopy; FTIR: Fourier transform infrared spectrometry; LSCM: Laser-scanning confocal microscope; OM: Optical microscopy; QCM: Quartz crystal microbalance; SERS: Surface-enhanced Raman spectroscopy; SEM: Electron scanning microscopy; TEM: Transmission electron microscopy; XPS: X-ray photoelectron spectroscopy; XRD: x-ray diffraction; UV-Vis: UV-Vis spectroscopy. The abbreviations of the materials are: GO: Graphene oxide; HUVEC: Human Umbilical Vein Endothelial Cells; mNSC: murine neural stem cell; MSC: Mesenchymal Stem Cell; MWCNT: Multi-walled carbon nanotubes; PCL: Poly(3-caprolactone); PLA: Polylactide; PLLA: Poly-L- lactide; PVA: Polyvinyl alcohol; PIEC: Pig iliac endothelial cells; RASMC: Rat aorta smooth muscle cells; SAP: Self-assembling peptides; SC: Rat Schwann cells; SF: Silk fibroin; VSMC: Vascular smooth muscle cells.

System	Reference
**Polymers**	**Al Rez, M. F., et al. (2017)**. "Tubular poly(ε-caprolactone)/chitosan nanofibrous scaffold prepared by electrospinning for vascular tissue engineering applications." *Journal of Biomaterials and Tissue Engineering 7(6): 427-436.* Scaffolds from Poly(ε-caprolactone) and Chitosan were fabricated by electrospinning and characterized with AFM, FITR, SEM, DSC and TGA. The authors showed that HUVEC cells are able to grow and proliferate on such scaffold, representing an alternative for blood vessel applications.
	**Chen, J., et al. (2015)**. "Biocompatible, Biodegradable, and Electroactive Polyurethane-Urea Elastomers with Tunable Hydrophilicity for Skeletal Muscle Tissue Engineering." *ACS Applied Materials and Interfaces 7(51): 28273-28285.* The presents a novel biomaterial with elastic and electroactive properties with the idea to control tissue regeneration by influencing cell behavior. The polyurethane-urea copolymers were suitable for adhesion and proliferation of C2C12 myoblast cells.
	**Chen, R., et al. (2010)**. "Preparation and characterization of coaxial electrospun thermoplastic polyurethane/collagen compound nanofibers for tissue engineering applications." *Colloids and Surfaces B: Biointerfaces 79(2): 315-325.* The authors present a scaffold based on nanofibers of collagen and thermoplastic polyurethane prepared by electrospinning. They characterized the scaffold with SEM, TEM, XPS, FITR and AFM (height images). The compatibility of the scaffolds was tested on PIEC cells.
**Collagen**	**Stylianou, A. and D. Yova (2013)**. "Surface nanoscale imaging of collagen thin films by Atomic Force Microscopy." *Materials Science and Engineering C 33(5): 2947-2957.* Since collagen is extensively used as biomaterial the authors characterized collagen thin films formed on different substrates with AFM. The structure of the collagen film could be modelled by spin coating and controlling the hydrodynamic flow. In this way, the authors could influence the orientation of the collagen films, which could be of importance for cell adhesion.
**Silk Fibroin**	**Farokhi, M., et al. (2014)**. "Structural and functional changes of silk fibroin scaffold due to hydrolytic degradation." *Journal of Applied Polymer Science 131(6).* The paper deals with the degradation of silk fibroin phosphate buffer saline after 12 weeks of incubation. Degradation has an impact on the biocompatibility of the fiber. Thus incubation changed the physical properties of the scaffold. Among the different experimental techniques, the authors use AFM to evaluate Young´s modulus of treated and untreated silk fibroin.
	**Pillai, M. M., et al. (2016)**. "Silk–PVA Hybrid Nanofibrous Scaffolds for Enhanced Primary Human Meniscal Cell Proliferation." *Journal of Membrane Biology 249(6): 813-822.* SF-PVA-based scaffolds are employed for attachment and proliferation of human meniscal cells. The authors compared different ratios and found that a 3:1 ratio of SF:PVA permitted the proliferation of the cells in comparison with pure SF-PVA nanofibers. Here AFM was just used to characterize the morphology of the scaffolds.
	**Zhang, K., et al. (2010)**. "Electrospun scaffolds from silk fibroin and their cellular compatibility." *Journal of Biomedical Materials Research - Part A 93(3): 976-983.* Scaffolds of silk fibroin were fabricated with electrospinning and characterized by SEM and AFM. The scaffolds promoted attachment and proliferation of PIECs, making them suitable for cardiovascular tissue engineering.
**Cellullose**	**Jia, B., et al. (2013)**. "Effect of microcrystal cellulose and cellulose whisker on biocompatibility of cellulose-based electrospun scaffolds." *Cellulose 20(4): 1911-1923.* The authors use micro- and nano-scaled cellulose particulates to improve the biocompatibility of scaffolds for vascular tissue engineering. The effect of MCC and CW on the biocompatibility of the scaffolds for VSMCs is tested. The scaffolds were characterized by FTIR, SEM, TEM and AFM (topography study).
**Peptides/Proteins**	**Babolmorad, G., et al. (2015)**. "Enhanced PC12 cells proliferation with self-assembled S-Layer proteins scaffolds." *Applied Biochemistry and Biotechnology 175(1): 223-231.*

	S-layer proteins (from *Bacillus coagulans* HN68) are used as a biocompatible alternative to synthetic materials. AFM was used to investigate S-layer crystallinity. The authors report that S-layers, with is micro -and nanopore structure, are a suitable scaffold for PC12 cells proliferation.
	**Silva, D., et al. (2013)**. "Synthesis and characterization of designed BMHP1-derived self-assembling peptides for tissue engineering applications." *Nanoscale 5(2): 704-718.*<br>In this work, the authors showed that SAPs scaffolds enhanced adhesion and proliferation of murine neural stem cell (mNSC). The characterization of the scaffold was very exhaustive: FTIR, XRD, rheometry, and AFM (which was used to elucidate the fiber nano-morphology).
**CNT/Graphene**	**Zhang, K., et al. (2016)**. "Aligned PLLA nanofibrous scaffolds coated with graphene oxide for promoting neural cell growth." *Acta Biomaterialia 37: 131-142.*<br>The authors combined the properties of GO and PLLA scaffolds. SCs growth was promoted on aligned PLLA scaffolds coated with GO. It also induced PC12 cell differentiation and neurite growth. The results are supported by a strong experimental study: AFM (fiber topography), LSCM, SEM, TEM, XPS.
	**Zhao, X., et al. (2015)**. "Investigation of polylactide/poly(ε-caprolactone)/multi-walled carbon nanotubes electrospun nanofibers with surface texture." *RSC Advances 5(120): 99179-99187.*<br>The authors investigate the influence of MWCNTs on the crystallization behavior, mechanical properties and compatibility of PLA/PCL nanofibers. SEM, tensile tests and AFM (nanoscale topography) were utilized to characterize the fibers, which were not toxic for mouse fibroblasts cells.
**Particles**	**Chen, C., et al. (2007)**. "Poly(lactic acid) (PLA) based nanocomposites - A novel way of drug-releasing." Biomedical Materials 2(4): L1-L4.<br>The authors show the viability of TiO_2-PLA doped with nanofibers with daunorubicin for cancer applications on leukemia K562 cells. Due to the high surface area of the nanocomposite, the scaffold can be seen as promising for drug delivery. The authors combine AFM and LSCM to show the assembly of daunorubicin in the scaffold structure.
	**Hung, H. S., et al. (2014).** "In vitro study of a novel nanogold-collagen composite to enhance the mesenchymal stem cell behavior for vascular regeneration." *PLoS ONE 9(8).*<br>The authors stated that composites based on collagen and gold nanoparticles promote the proliferation and migration of MSCs. In addition, the composites stimulate the endothelial cell differentiation. The experimental techniques used were: UV-Vis, SERS, SEM, FM, and AFM (to study the surface topography). The results are promising for vascular regeneration applications.
**Patterns**	**Li, M., et al. (2005)**. "Comparison of selective attachment and growth of smooth muscle cells on gelatin- and fibronectin-coated micropatterns." Journal of Nanoscience and Nanotechnology 5(11): 1809-1815.<br>The authors built micro-patterned scaffolds of gelatin and fibronectin with the purpose to study the behaviour of RASMCs. The patterns were fabricated using the layer by layer technique. Cell growing was affected by the shape of the patterns. CA, QCM, FM, and AFM (pattern topography) were used to characterize the system.
	**Marszalek, J. E., et al. (2013)**. "2.5D constructs for characterizing phase separated polymer blend surface morphology in tissue engineering scaffolds." *Journal of Biomedical Materials Research - Part A 101 A(5): 1502-1510.*<br>In this work, the authors expose PCL-PDLLA blends to NaCl in order to influence phase separation in the blends. The work has a technical contribution since the authors propose a new 2.5D approach to characterize the samples with AFM and OM, as well as to investigate cell adhesion.
